# Galectin-3 and Galectin-9 May Differently Regulate the Expressions of Microglial M1/M2 Markers and T Helper 1/Th2 Cytokines in the Brains of Genetically Susceptible C57BL/6 and Resistant BALB/c Mice Following Peroral Infection With *Toxoplasma gondii*

**DOI:** 10.3389/fimmu.2018.01648

**Published:** 2018-07-31

**Authors:** Jinfeng Liu, Shiguang Huang, Fangli Lu

**Affiliations:** ^1^Department of Parasitology, Zhongshan School of Medicine, Sun Yat-sen University, Guangzhou, China; ^2^Key Laboratory of Tropical Disease Control of Ministry of Education, Sun Yat-sen University, Guangzhou, China; ^3^School of Stomatology, Jinan University, Guangzhou, China

**Keywords:** toxoplasmic encephalitis, galectins, microglial M1/M2 markers, *T. gondii* microneme proteins, mice

## Abstract

Toxoplasmic encephalitis (TE), an opportunistic infection, is a severe health problem in immunocompromised patients. Previous studies have revealed that C57BL/6 mice are susceptible and BALB/c mice are resistant to TE. To investigate the mechanisms involved in the immunopathogenesis of TE in susceptible C57BL/6 and resistant BALB/c mice, both strains of mice were perorally infected with the Prugniuad (Pru) strain of *Toxoplasma gondii*. Our results showed that compared with BALB/c mice, C57BL/6 mice infected with *T. gondii* Pru strain had more severe brain histopathological damage, and higher mRNA expression levels of tachyzoite-specific surface antigen 1, bradyzoite-specific antigen 1, interferon gamma (IFNγ), interleukin (IL)-10, arginase1 (Arg1) (M2 marker), galectin (Gal)-3, Gal-9, *T. gondii* microneme protein 1 (TgMIC1), TgMIC4, and TgMIC6 during the course of infection by using quantitative real-time reverse transcription-polymerase chain reaction. Further analysis displayed that BALB/c mice showed higher numbers of microglial cells and higher levels of IL-1β, inducible nitric oxide synthase (iNOS) (M1 marker), and chitinase-3-like protein 3 (Ym1) (M2 marker) in the early infective stage [at day 14 or 35 post infection (p.i.)] compared with C57BL/6 mice, whereas C57BL/6 mice showed higher numbers of microglial cells and higher levels of IL-10, iNOS (M1 marker), and Ym1 (M2 marker) at days 35, 50, or 70 p.i. compared with BALB/c mice. Correlation analysis showed that significant positive correlations existed between Gal-3 and IL-4/IL-10/iNOS/Ym1 and between Gal-9 and IL-4/Ym1 in C57BL/6 mice; between Gal-3 and IFNγ/Arg1 and between Gal-9 and IFNγ/Arg1 in BALB/c mice. Together, our data demonstrated that different Gal-3 and Gal-9 expressions as well as different positive correlations were found between Gal-3 and T helper 1 (Th1)/Th2/M1/M2 cytokines or between Gal-9 and Th1/Th2/M2 cytokines in the brains of *T. gondii* Pru strain-infected C57BL/6 and BALB/c mice.

## Introduction

*Toxoplasma gondii*, a pathogen of medical and veterinary importance, is an obligate intracellular protozoan parasite that has a global distribution and can infect almost any warm-blooded vertebrate (1). *T. gondii* infection in the immunocompetent individual is effectively controlled by a vigorous immune response (2); however, the infection can cause toxoplasmic encephalitis (TE), a life-threatening disease in immunocompromised patients (3). Although all mice lineages develop a strong T helper 1 (Th1) immune response to *T. gondii* infection (4), the immune response to the parasite infection in the brains can be drastically different between genetically resistant mice (e.g., BALB/c mice) and that of susceptible mice (e.g., C57BL/6 mice) (5). During the late stage of infection, resistant mouse strain establishes a latent chronic infection, while susceptible strain spontaneously develops necrotizing TE (6). So far, the mechanisms behind the differences between the two strains of mice during the development of TE are not fully understood.

It has been proposed that *T. gondii* utilizes innate immune cells such as macrophages to migrate to immunoprivileged sites such as the central nervous system (CNS) to establish chronic infection (7). Macrophages are generally categorized into two distinct subsets as either classically activated (M1) or alternatively activated (M2). M1 type macrophages, characterized by CD86 expression, can release high levels of pro-inflammatory markers such as monocyte chemotactic protein-1β, inducible nitric oxide synthase (iNOS), interleukin (IL)-6, and tumor necrosis factor alpha (TNFα) (8). M2 macrophages can produce a large amount of IL-10, chitinase-3-like protein 3 (Ym1), macrophage and granulocyte inducer-form 1, and arginase1 (Arg1) and play important roles in the protection of the host by decreasing inflammation and promoting tissue repair (9, 10). During *T. gondii* infection, Th1 cells produce cytokines such as interferon gamma (IFNγ) to activate macrophages and cytotoxic T lymphocytes, while Th2 cells secrete cytokines such as IL-4 to induce humoral type immune responses (11, 12). IFNγ-activated microglial cells significantly upregulate iNOS and produce nitric oxide (NO), which can inhibit intracellular *T. gondii* replication (13).

Galectins belong to the family of β-galactoside-binding lectins, which are known to regulate a number of pathways that involve in apoptosis (14), immune tolerance, inflammation (15), and cell adhesion (16). Currently, 15 members of the galectin family have been identified in mammals; some members are widely distributed in different cells and tissue types, while others are more selectively expressed (17). The major galectins expressed in the CNS are galectin (Gal)-1, Gal-3, Gal-4, Gal-8, and Gal-9 (18). Under normal physiological conditions, galectins maintain CNS homeostasis, while in neuronal diseases and experimental neuroinflammatory disease models, galectins may serve as extracellular mediators or intracellular regulators in controlling the inflammatory response or conferring the remodeling capacity in damaged CNS tissues (18). So far, the roles of galectins in TE remains poorly understood.

Apicomplexan parasites such as *T. gondii* and *Plasmodium* spp. utilize apical complex organelles consisting of dense granules, rhoptries, and micronemes to deploy for the release (egress), attachment, and invasion of host cells, as well as the establishment of the parasitophorous vacuole (19). *T. gondii* microneme proteins (TgMICs) are secreted by micronemes upon contact with host cells and play important roles in *T. gondii* motility, invasion, intracellular survival, and egress from host cells (20). TgMIC6 and TgMIC8 genes are expressed in the rapidly dividing tachyzoites, whereas TgMIC7 and TgMIC9 genes are predominantly expressed in the slowly dividing encysted bradyzoites (21). TgMIC1 and TgMIC4 can bind to host cells, while TgMIC6 serves as an escorter for two soluble adhesins TgMIC1 and TgMIC4 and along with adhesins can establish a molecular bridge between the host and parasites (22). So far, limited data are available about the role of TgMICs in the immune response to *T. gondii* infection.

Based on the relationship between galectins and brain diseases, this study was designed to compare the expressions of galectins, microglial activation markers (M1 and M2 phenotypes), TgMICs, and Th1 and Th2 cytokines between C57BL/6 and BALB/c mice infected with *T. gondii* Pru strain. We found that significant positive correlations existed between Gal-3 and Th1/Th2/M1/M2 cytokines as well as between Gal-9 and Th1/Th2/M2 cytokines in C57BL/6 or BALB/c mice after *T. gondii* Pru strain infection.

## Materials and Methods

### Mice, Parasites, and Experimental Infections

This experimental study and all administrations were reviewed and approved by the Ethical Committee of Animal Experiments at Sun Yat-sen University.

Female 6- to 8-week-old C57BL/6 and BALB/c mice were purchased from the Experimental Animal Center at Sun Yat-sen University (Guangzhou, China), and 20 mice were used per each group. All animals were housed under specific-pathogen-free conditions in the animal facility at Sun Yat-sen University. Mice were infected *via* oral route with eight cysts of *T. gondii* Pru strain prepared from the brain of chronically infected mice. To establish a chronic infection by controlling the proliferation of tachyzoites during acute stage, mice were treated with sulfadiazine (Sigma-Aldrich, Shanghai, China) in the drinking water as described previously (23).

### Histopathology

Mice infected with *T. gondii* Pru strain were euthanatized by CO_2_ asphyxiation at 14, 35, 50, and 70 days post infection (p.i.), and their brains were harvested and immediately fixed in 10% buffered natural formaldehyde (Guangzhou Chemical Reagent Factory, China) for over 48 h. The paraffin-embedded tissues from each mouse were sectioned at 5 µm and prepared for hematoxylin and eosin (Sigma-Aldrich, Shanghai, China) staining. The histopathological changes of brains from each group were determined under 200× magnification in three noncontiguous sections from four mice, and histopathological scores were given based on previously described criteria (23) with some modifications. In brief, the histological changes were scored semi-quantitatively as 1, 2, 3, and 4 (e.g., normal, mild inflammation, moderate inflammation and necrosis, and severe inflammation and necrosis, respectively).

### Immunohistochemical Staining

The paraffin-embedded brain sections (6-µm) were deparaffinized and rehydrated in distilled water. Heat-induced antigen retrieval was carried out in an 800-W microwave oven for 30 min. Sections were treated with 3% hydrogen peroxide in methanol for 10 min at 37°C, and then incubated in 10% normal goat serum with 1% bovine serum albumin (Sigma-Aldrich, Shanghai, China) in PBS (pH 7.4) for 10 min at room temperature to block nonspecific binding. After washing with PBS, sections were incubated with rabbit anti-Iba1 (1:200 dilutions) (Wako Pure Chemical Industries, Osaka, Japan), rabbit anti-Gal-9 (1:200 dilutions) (Boster Biological Technology, Wuhan, China), and mouse anti-Gal-3 (1:200 dilutions) (R&D Systems, Minneapolis, MN, USA) overnight at 4°C. Those sections incubated with secondary antibodies alone were used as isotype controls. Immunohistochemical staining was then performed with a streptavidin–biotin–peroxidase complex kit and developed with diaminobenzidine tetrahydrochloride (Zhongshan Golden Bridge Technology, Beijing, China). The sections were counterstained with hematoxylin and positive cells were identified by dark-brown staining under light microscopy.

### Morphometric Analysis

Serial sections from the brains were immunostained with anti-Iba1. A total of three mice were analyzed in each time point, and four sections per animal were selected for counting of positive cells. In every brain section, the microglial cells expressing Iba1 markers were captured with a digital microscopy under 400× magnification and the numbers of Iba1-positive cells in the brains (0.015066 mm^2^ tissue section) were determined by Image-Pro Plus (Image Z1 software, Media Cybernetics, MD, USA), and the density of positive cells was expressed as the number of cells per square millimeter.

### Selection of Galectins

Gal-1, Gal-3, Gal-7, Gal-8, and Gal-9 are known to be relevant to brain diseases (18). Therefore, in this study, the specific expression pattern of these five galectins was examined.

### Determination of mRNA Expression Using Quantitative Real-Time Reverse Transcription-Polymerase Chain Reaction (qRT-PCR)

Total RNA was extracted from about 100 mg of mouse brain tissues from each group using a RNA Extraction Kit (TaKaRa, Shiga, Japan) as per the manufacturer’s protocol. RNA amount was determined by measuring the ratio of absorbance at 260 and 280 nm using a NanoDrop ND-1000 spectrophotometer (NanoDrop Technologies). First-strand cDNA was constructed from 1.0 µg of total RNA with oligo (dT) as primers using a PrimeScript 1st Strand cDNA Synthesis Kit (TaKaRa, Shiga, Japan). To determine tissue mRNA levels of cytokines (IL-1β, IFNγ, IL-4, IL-10, iNOS, Arg1, and Ym-1), galectins (Gal-1, Gal-3, Gal-4, Gal-8, and Gal-9), TgMICs (TgMIC1, TgMIC4, TgMIC6, TgMIC3, and TgMIC8), β-actin, actin of the ME49 strain of *T. gondii, T. gondii* tachyzoite-specific surface antigen 1 (SAG1), and *T. gondii* bradyzoite-specific antigen 1 (BAG1), qRT-PCR measurements were performed using SYBR Green QPCR Master Mix (TaKaRa, Shiga, Japan). Primers are listed in Table 1. Briefly, a total of 10 µl reaction mixture contained 5.0 µl of SYBR^®^ Premix Ex TaqTM (2×), 0.5 µl of each primer (10 pM), 3.0 µl of dH_2_O, and 1.0 µl of cDNA (0.2 µg/µl). Amplification was pre-denaturized for 30 s at 95°C, followed by 43 cycles of 5 s at 95°C and 20 s at 60°C with a LightCycler^®^ 480 instrument (Roche Diagnostics, USA). The mRNA expression levels of cytokines, SAG1, and BAG1 were normalized to that of mouse housekeeping gene, β-actin, and the mRNA levels of TgMICs were normalized to that of *T. gondii* housekeeping gene (actin of *T. gondii* ME49 strain). The results were expressed as fold change compared with uninfected controls.

**Table 1:** Primer sequences of genes used for quantitative real-time reverse transcription-polymerase chain reaction assays.

Genes	Forward primer (5′→3′)	Reverse primer (5′→3′)	Reference/accession
IL-1β	AATGACCTGTTCTTTGAAGTTGA	TGATGTGCTGCTGCGAGATTTGAAG	(24)
IFNγ	GGAACTGGCAAAAGGATGGTGAC	GCTGGACCTGTGGGTTGTTGAC	(25)
IL-4	ACAGGAGAAGGGACGCCAT	GAAGCCCTACAGACGAGCTCA	(26)
IL-10	AGCCGGGAAGACAATAACTG	CATTTCCGATAAGGCTTGG	(25)
iNOS	GTTCTCAGCCCAACAATACAAGA	GTGGACGGGTCGATGTCAC	(27)
Arg1	CTCCAAGCCAAAGTCCTTAGAG	AGGAGCTATCATTAGGGACATC	(27)
Ym1	AGAAGGGAGTTTCAA ACCTGGT	GTCTTGCTCATGTGTGTAAGTGA	(27)
Gal-1	CGCCAGCAACCTGAATC	GTCCCATCTTCCTTGGTGTTA	(28)
Gal-3	GCTACTGGCCCCTTTGGT	CCAGGCAAGGGCATATCGTA	(29)
Gal-4	CAACCCTCCACAGATGAACACCTT	TCCAGCGTGTCTACCATTTGGAAT	(30)
Gal-8	GGGTGGTGGGTGGAACTG	GCCTTTGAGCCCCCAATATC	(31)
Gal-9	GAGCTTTGCTTCCTGGTACAGA	CGGTGTGAGTACTGTACAAAGAAGT	(29)
β-actin	TGGAATCCTGTGGCATCCATGAAAC	TAAAACGCAGCTCAGTAACAGTCCG	(25)
TgMIC1	GCGAATTTCCTTGATGGATT	GTAGTCGAGGACAACAGCGA	XM_002368490.1
TgMIC3	AGCCATCACACACACACCTT	ATGCACAGAAACGCACTCTC	XM_002369792.1
TgMIC4	CCTGCAAGGCTTCACTGATA	CTATTGTGGGAGCCCTTGAT	XM_002369565.1
TgMIC6	CGCCAGATGCAGTACAGAGT	GCGTCGATTGTCGCTATAAA	XM_002370595.1
TgMIC8	GTAAAGGCGAGGTCGAAGAC	GTACTGCGGGAAAGGATGAT	XM_002366938.1
*T. gondii* ME49 actin	ATTATGAAGTGCGACGTGGA	TGATCTTCATGGTGGAAGGA	XM_002369622.1

### Statistical Analysis

Results of experimental studies were reported as mean ± SD. Statistical analysis of the data was performed by the Wilcoxon rank sum test and one-way ANOVA followed by Bonferroni’s multiple comparison tests using SPSS software for windows (version 19.0; SPSS, Inc., IL, USA). Pearson’s correlation coefficient was used to analyze correlations between the levels of cytokines and galectins. All graphs were performed using GraphPad Prism software (version 5.0). A value of *P* < 0.01 was considered significant for correlation analysis, while a value of *P* < 0.05 was considered significant for other statistical analysis.

## Results

### Comparison of Histopathology and Parasite Burdens in the Brains of *T. gondii*-Susceptible C57BL/6 and *T. gondii*-Resistant BALB/c Mice

**Figure 1:**
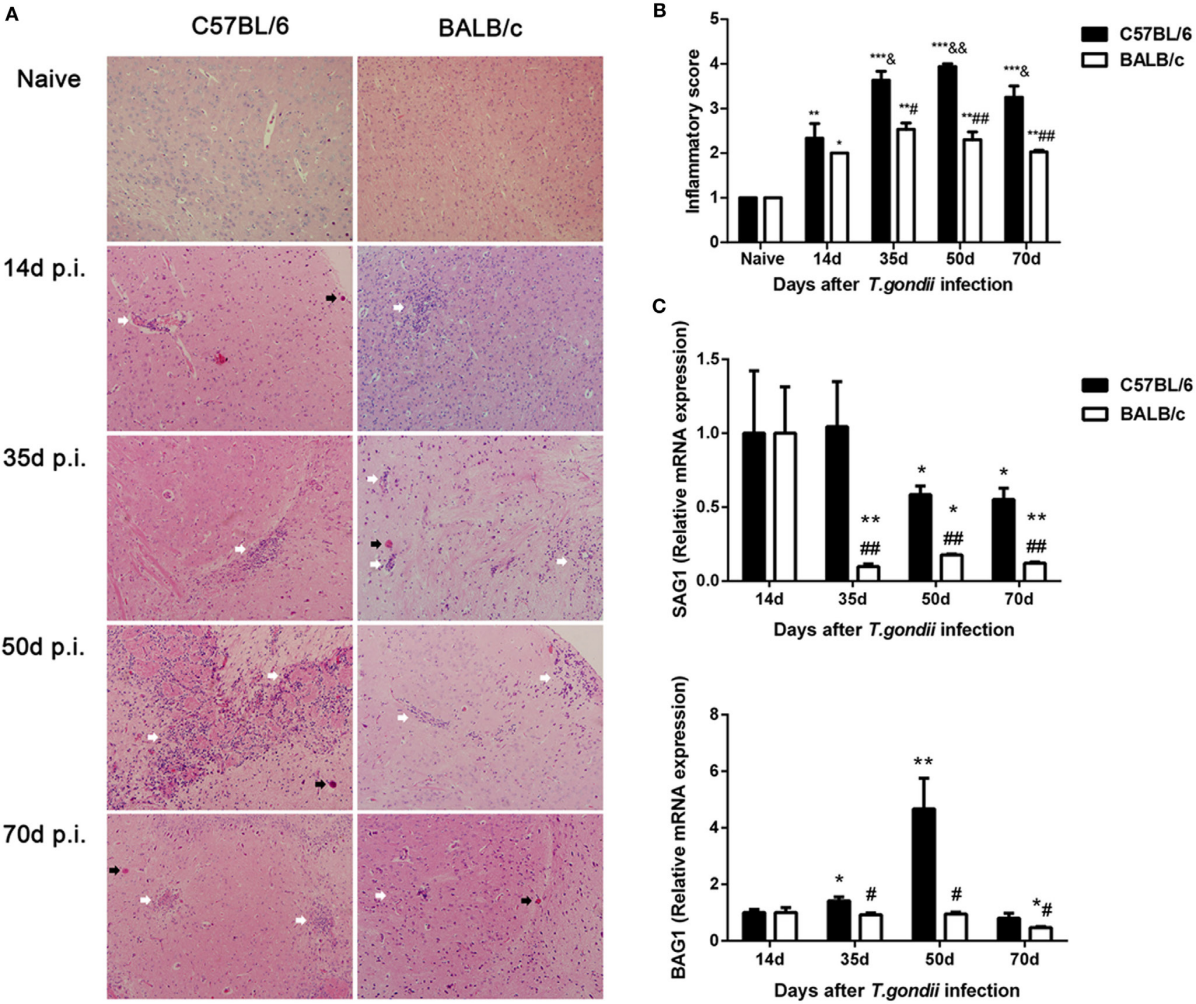
Histopathological changes and parasite burdens in the brains of *Toxoplasma gondii* Pru strain-infected C57BL/6 and BALB/c mice. **(A)** Histopathological changes in the brains at days 14, 35, 50, and 70 post infection (p.i.). Cysts were indicated with black arrow heads and inflammatory cell infiltrates were indicated with white arrow heads. Original magnification 200×; hematoxylin and eosin stain. **(B)** Histopathological score analysis at days 14, 35, 50, and 70 p.i. Data are represented as mean ± SEM. Significant differences between groups are analyzed by the Wilcoxon rank sum test. *P < 0.05, **P < 0.01, and ***P < 0.001 vs naive; ^&^*P* < 0.05 and ^&&^*P* < 0.01 vs 14 days p.i.; ^#^*P* < 0.05 and ^##^*P* < 0.01 vs C57BL/6 mice. **(C)** Parasite burdens in the brains at days 14, 35, 50, and 70 p.i. Relative mRNA expressions of surface antigen 1 (SAG1) and bradyzoite-specific antigen 1 (BAG1) were detected by using quantitative real-time reverse transcription-polymerase chain reaction. Transcript level at day 14 p.i. was taken as 1. Values are means from triplicate measurements, and data are presented as mean ± SD. *P < 0.05 and **P < 0.01 vs naive; ^#^*P* < 0.05 and ^##^*P* < 0.01 vs C57BL/6 mice. There were four mice per group. The data shown are representative of those from two different experiments.

Histological observation showed that control sections of the brains from uninfected C57BL/6 and BALB/c mice had no obvious inflammations or structural abnormalities. The brains of *T. gondii* Pru strain-infected C57BL/6 mice showed moderate-to-severe inflammation, diffuse inflammatory cellular infiltration, necrotic focus, and tissue structural damages at days 14, 35, 50, and 70 p.i., while the brains of infected BALB/c mice showed limited infiltration of inflammatory cells at the aforementioned times (Figure 1A). Semi-quantitative analysis of the severity of inflammation and necrosis in the brain sections of the two strains of mice were performed. Compared with uninfected controls, the pathological severity scores of brains were significantly increased in both C57BL/6 and BALB/c mice at days 14, 35, 50, and 70 p.i. Compared with BALB/c mice, the histopathological scores in the brains of C57BL/6 mice were significantly higher at days 35 (*P* < 0.05), 50 (*P* < 0.01), and 70 (*P* < 0.01) p.i. (Figure 1B).

Stage conversion between tachyzoite and bradyzoite forms is associated with stage specific antigen expression. In this study, the mRNA expression levels of tachyzoite-specific SAG1 and bradyzoite-specific BAG1 in the brains of C57BL/6 and BALB/c mice infected with *T. gondii* Pru strain were detected by using qRT-PCR and the transcript. Levels of SAG1 and BAG1 were relative to day 14 p.i. (e.g., the relative transcript level at day 14 p.i. = 1.0). Compared with day 14 p.i., the SAG1 levels in the brains of both C57BL/6 and BALB/c mice were significantly decreased at days 35, 50, and 70 p.i. The BAG1 levels in the brains of C57BL/6 mice were significantly elevated at days 35 and 70 p.i., while the BAG1 level in BALB/c mice was significantly reduced at day 70 p.i. Compared with BALB/c mice, both SAG1 and BAG1 levels were significantly higher in the brains of C57BL/6 mice at days 35, 50, and 70 p.i. (*P* < 0.01 and *P* < 0.05, respectively) (Figure 1C).

### Comparison of Microglial Cells in the Brains of *T. gondii*-Susceptible C57BL/6 and *T. gondii*-Resistant BALB/c Mice

**Figure 2:**
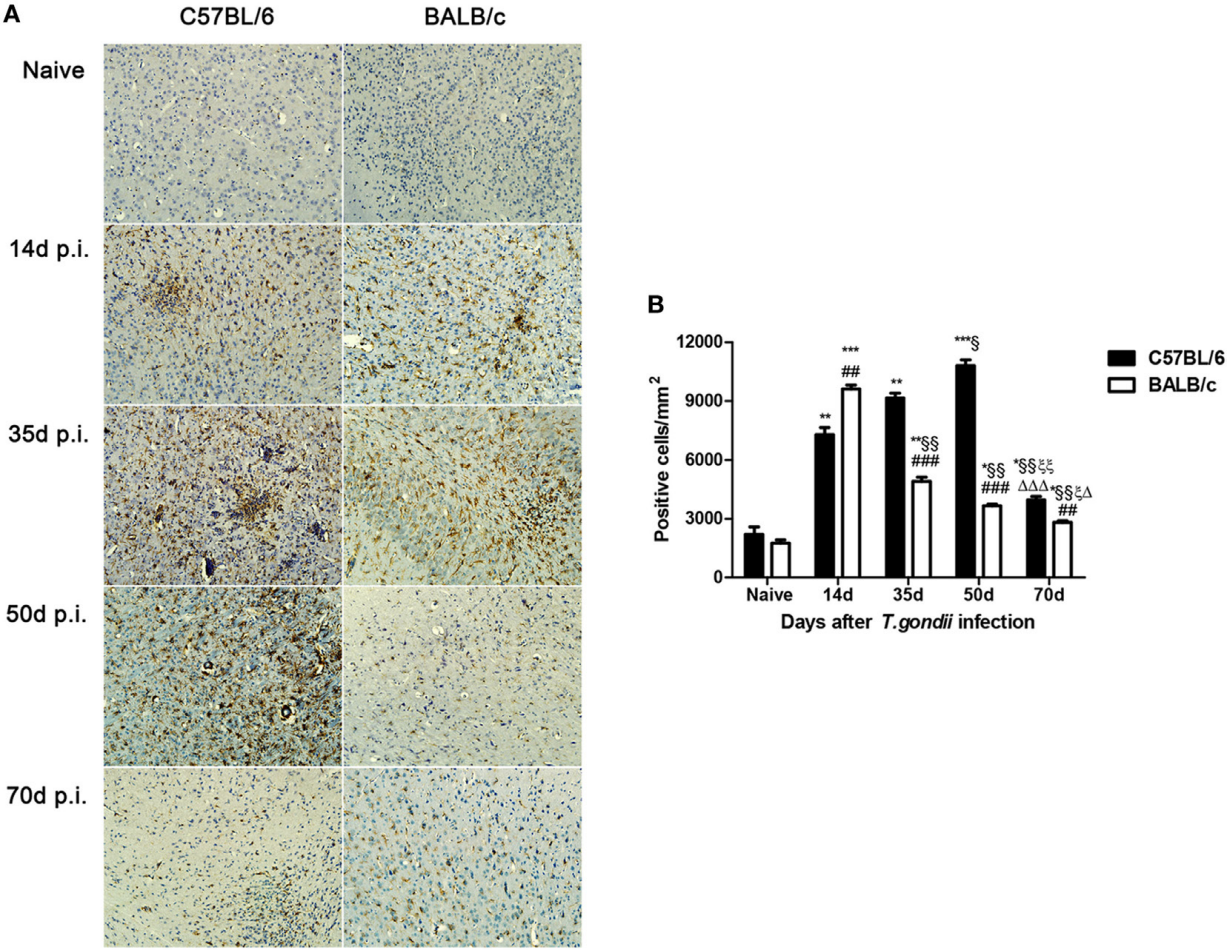
Expression of activated microglial marker Iba1 in the brains of *Toxoplasma gondii* Pru strain-infected C57BL/6 and BALB/c mice. **(A)** Immunohistochemistry for Iba1 in the brains of uninfected mice, and mice infected with *T. gondii* Pru strain at days 14, 35, 50, and 70 post infection (p.i.). Original magnification 200×. **(B)** Quantitative analysis of Iba1-positive microglia. The density of positive cells was expressed as the number of cells per square millimeter. Data are presented as means ± SD; experiments were performed with three mice per group. *P < 0.05, **P < 0.01, and ***P < 0.001 vs Naive; ^§^*P* < 0.05 and ^§§^*P* < 0.01 vs 14 days p.i.; ^ξ^*P* < 0.05 and ^ξξ^*P* < 0.01 vs 35 days p.i.; ^Δ^*P* < 0.05 and ^ΔΔΔ^*P* < 0.001 vs 50 days p.i.; ^##^*P* < 0.01 and ^###^*P* < 0.001 vs C57BL/6 mice.

A few Iba1-positive microglial cells were observed in the sections of brains of uninfected C57BL/6 and BALB/c mice. However, a large number of activated microglial cells were observed in the brains of both *T. gondii* Pru strain-infected C57BL/6 and BALB/c mice; the majority of activated microglial cells were ameboid shape with thickened and retracted branches (Figure 2A). Quantitative analysis of Iba1 staining showed that, compared with uninfected controls, the numbers of Iba1-positive microglial cells in the brains of both C57BL/6 and BALB/c mice were significantly increased at days 14, 35, 50, and 70 p.i. However, compared with BALB/c mice, the microglial cell numbers in the brains of C57BL/6 mice were significantly higher at days 35 (*P* < 0.001), 50 (*P* < 0.001), and 70 (*P* < 0.01) p.i. (Figure 2B).

### Comparison of mRNA Levels of M1/M2 Markers in the Brains of *T. gondii*-Susceptible C57BL/6 and *T. gondii*-Resistant BALB/c Mice

**Figure 3:**
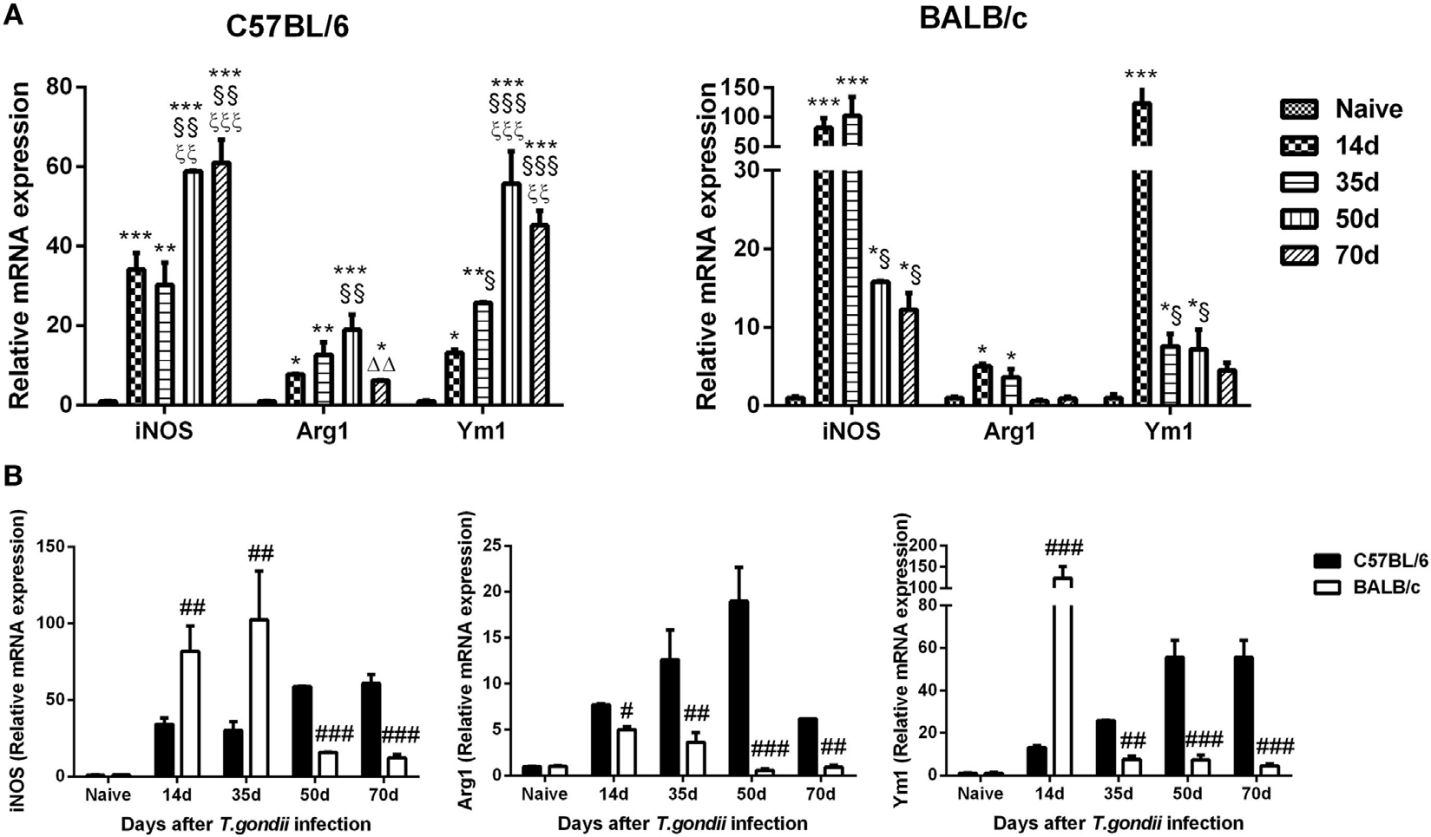
Relative mRNA expressions of inducible nitric oxide synthase (iNOS), arginase1 (Arg1), and Ym1 in the brain tissues of *Toxoplasma gondii* Pru strain-infected C57BL/6 and BALB/c mice were detected by using quantitative real-time reverse transcription-polymerase chain reaction. **(A)** iNOS, Arg1, and Ym1 expressions in the brains of C57BL/6 and BALB/c mice. **(B)** Comparison of iNOS, Arg1, and Ym1 levels in the brains of C57BL/6 and BALB/c mice. Values are means from triplicate measurements, and data are presented as mean ± SD. There were four mice per group. The data shown are representative of those from two different experiments. *P < 0.05, **P < 0.01, and ***P < 0.001 vs naive; ^§^*P* < 0.05; ^§§^*P* < 0.01, and ^§§§^*P* < 0.001 vs 14 days post infection (p.i.); ^ξξ^*P* < 0.01 and ^ξξξ^*P* < 0.001 vs 35 days p.i.; ^ΔΔ^*P* < 0.01 vs 50 days p.i.; ^#^*P* < 0.05, ^##^*P* < 0.01, and ^###^*P* < 0.001 vs C57BL/6 mice.

The mRNA levels of M1 marker (iNOS) and M2 marker (Arg1 and Ym1) in the brains of *T. gondii* Pru strain-infected C57BL/6 and BALB/c mice were examined. Compared with uninfected controls, iNOS levels were significantly increased in the brains of both C57BL/6 and BALB/c mice at days 14, 35, 50, and 70 p.i.; Arg1 and Ym1 levels were significantly increased in C57BL/6 mice at days 14, 35, 50, and 70 p.i., and significantly increased in BALB/c mice at days 14 and 35 p.i. (Figure 3A). Compared with BALB/c mice, there were significantly lower iNOS levels at days 14 and 35 p.i. (*P* < 0.01); while there were significantly higher iNOS levels at days 50 and 70 p.i. (*P* < 0.001), significantly higher Arg1 levels at days 14 (*P* < 0.05), 35 (*P* < 0.01), 50 (*P* < 0.001), and 70 (*P* < 0.01) p.i., and significantly lower Ym1 level at day 14 p.i. (*P* < 0.001) and significantly higher Ym1 levels at days 35 (*P* < 0.01), 50 (*P* < 0.001), and 70 (*P* < 0.001) p.i. in the brains of C57BL/6 mice (Figure 3B).

### Comparison of mRNA Levels of Th1/Th2 Cytokines in the Brains of *T. gondii*-Susceptible C57BL/6 and *T. gondii*-Resistant BALB/c Mice

**Figure 4:**
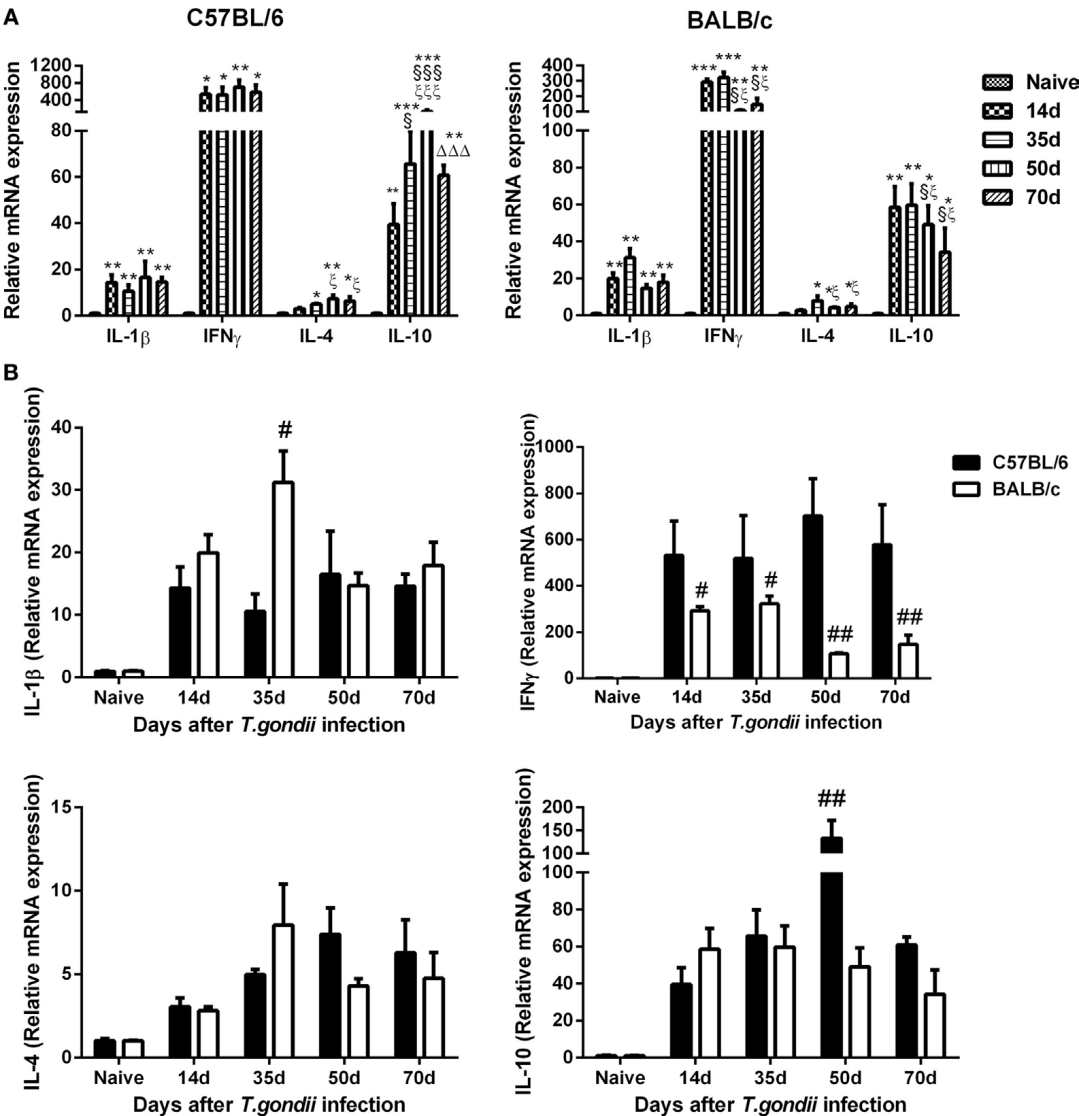
Relative mRNA expressions of interleukin (IL)-1β, interferon gamma (IFNγ), IL-4, and IL-10 in the brain tissues of *Toxoplasma gondii* Pru strain-infected C57BL/6 and BALB/c mice were detected by using quantitative real-time reverse transcription-polymerase chain reaction. **(A)** IL-1β, IFNγ, IL-4, and IL-10 expressions in the brains of C57BL/6 and BALB/c mice. **(B)** Comparison of IL-1β, IFNγ, IL-4, and IL-10 levels in the brains of C57BL/6 and BALB/c mice. Values are means from triplicate measurements, and data are presented as mean ± SD. There were four mice per group. The data shown are representative of those from two different experiments. *P < 0.05, **P < 0.01, and ***P < 0.001 vs naive; ^§^*P* < 0.05 and ^§§§^*P* < 0.001 vs 14 days post infection (p.i.); ^ξ^*P* < 0.05 and ^ξξξ^*P* < 0.001 vs 35 days p.i.; ^ΔΔΔ^*P* < 0.001 vs 50 days p.i.; ^#^*P* < 0.05 and ^##^*P* < 0.01 vs C57BL/6 mice.

Compared with uninfected controls, the levels of IL-1β, IFNγ, and IL-10 were significantly increased in the brains of both C57BL/6 and BALB/c mice at days 14, 35, 50, and 70 p.i.; IL-4 levels were significantly increased in both *T. gondii* Pru strain-infected C57BL/6 and BALB/c mice at days 35, 50, and 70 p.i. (Figure 4A). Compared with BALB/c mice, significantly lower IL-1β level at day 35 p.i. (*P* < 0.05), significantly higher IL-10 level at day 50 p.i. (*P* < 0.01), and significantly higher IFNγ levels at days 14 (*P* < 0.05), 35 (*P* < 0.05), 50 (*P* < 0.01), and 70 (*P* < 0.01) p.i. were detected in the brains of C57BL/6 mice (Figure 4B).

### Comparison of mRNA Levels of Galectins in the Brains of *T. gondii*-Susceptible C57BL/6 and *T. gondii*-Resistant BALB/c Mice

**Figure 5:**
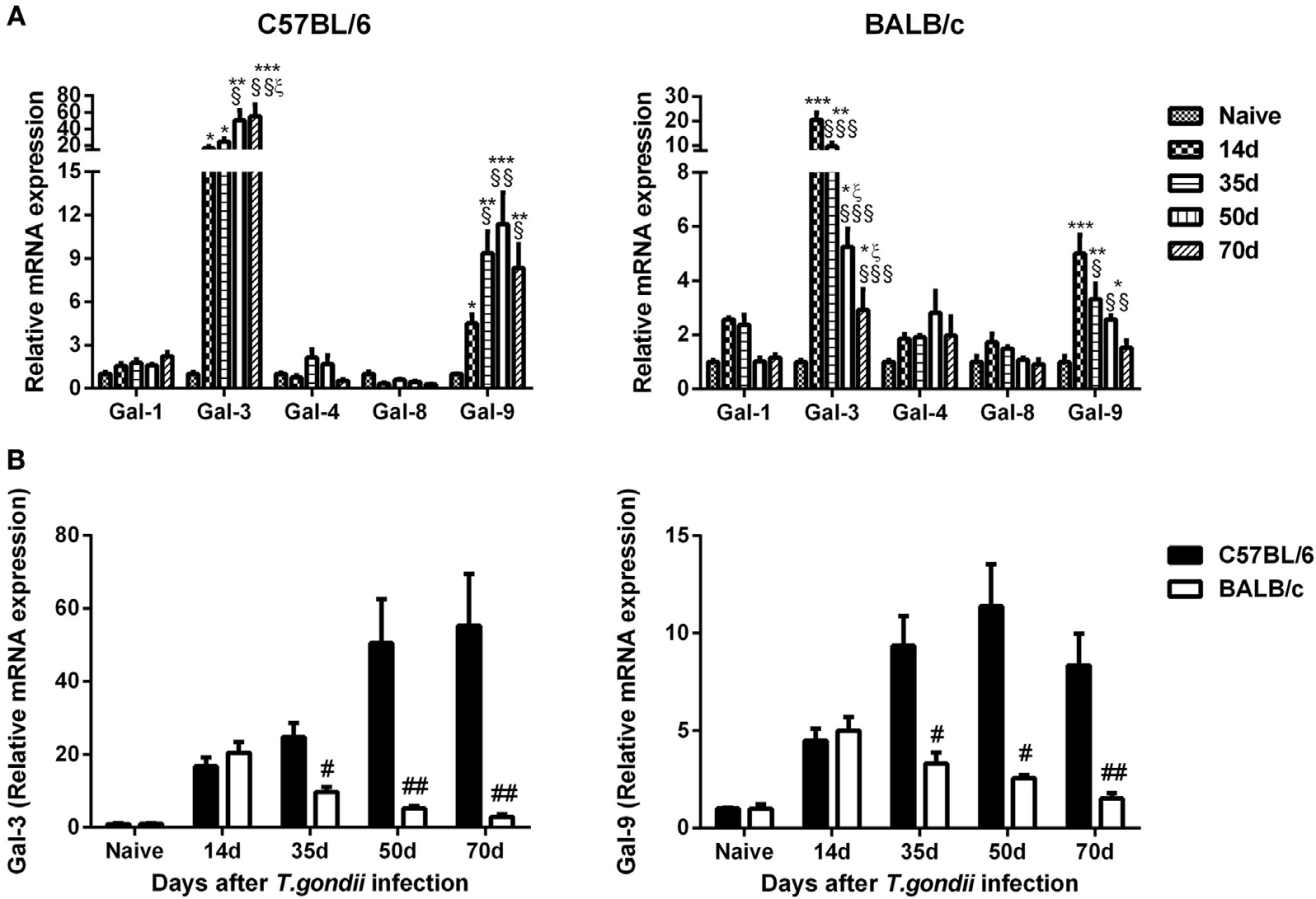
Relative mRNA expressions of Gal-1, Gal-3, Gal-4, Gal-8, and Gal-9 in the brain tissues of *Toxoplasma gondii* Pru strain-infected C57BL/6 and BALB/c mice were detected by using quantitative real-time reverse transcription-polymerase chain reaction. **(A)** Galectin expressions in the brains of C57BL/6 and BALB/c mice. **(B)** Comparison of Gal-3 and Gal-9 levels in the brains of C57BL/6 and BALB/c mice. Values are means from triplicate measurements, and data are presented as mean ± SD. There were four mice per group. The data shown are representative of those from two different experiments. *P < 0.05, **P < 0.01, and ***P < 0.001 vs naive; ^§^*P* < 0.05, ^§§^*P* < 0.01, and ^§§§^*P* < 0.001 vs 14 days post infection (p.i.); ^ξ^*P* < 0.05 vs 35 days p.i.; ^#^*P* < 0.05 and ^##^*P* < 0.01 vs C57BL/6 mice.

Compared with uninfected controls, Gal-3 expression levels were significantly increased in the brains of both *T. gondii* Pru strain-infected C57BL/6 and BALB/c mice at days 14, 35, 50, and 70 p.i.; Gal-9 levels were significantly increased in both C57BL/6 and BALB/c mice at days 14, 35, and 50 p.i., and significantly increased in C57BL/6 mice at day 70 p.i. (Figure 5A). Compared with BALB/c mice, there were significantly higher levels of Gal-3 and Gal-9 in the brains of C57BL/6 mice at days 35 (*P* < 0.05), 50 (*P* < 0.01 and *P* < 0.05, respectively), and 70 (*P* < 0.01) p.i. (Figure 5B).

### Comparison of mRNA Levels of TgMICs in the Brains of *T. gondii*-Susceptible C57BL/6 and *T. gondii*-Resistant BALB/c Mice

**Figure 6:**
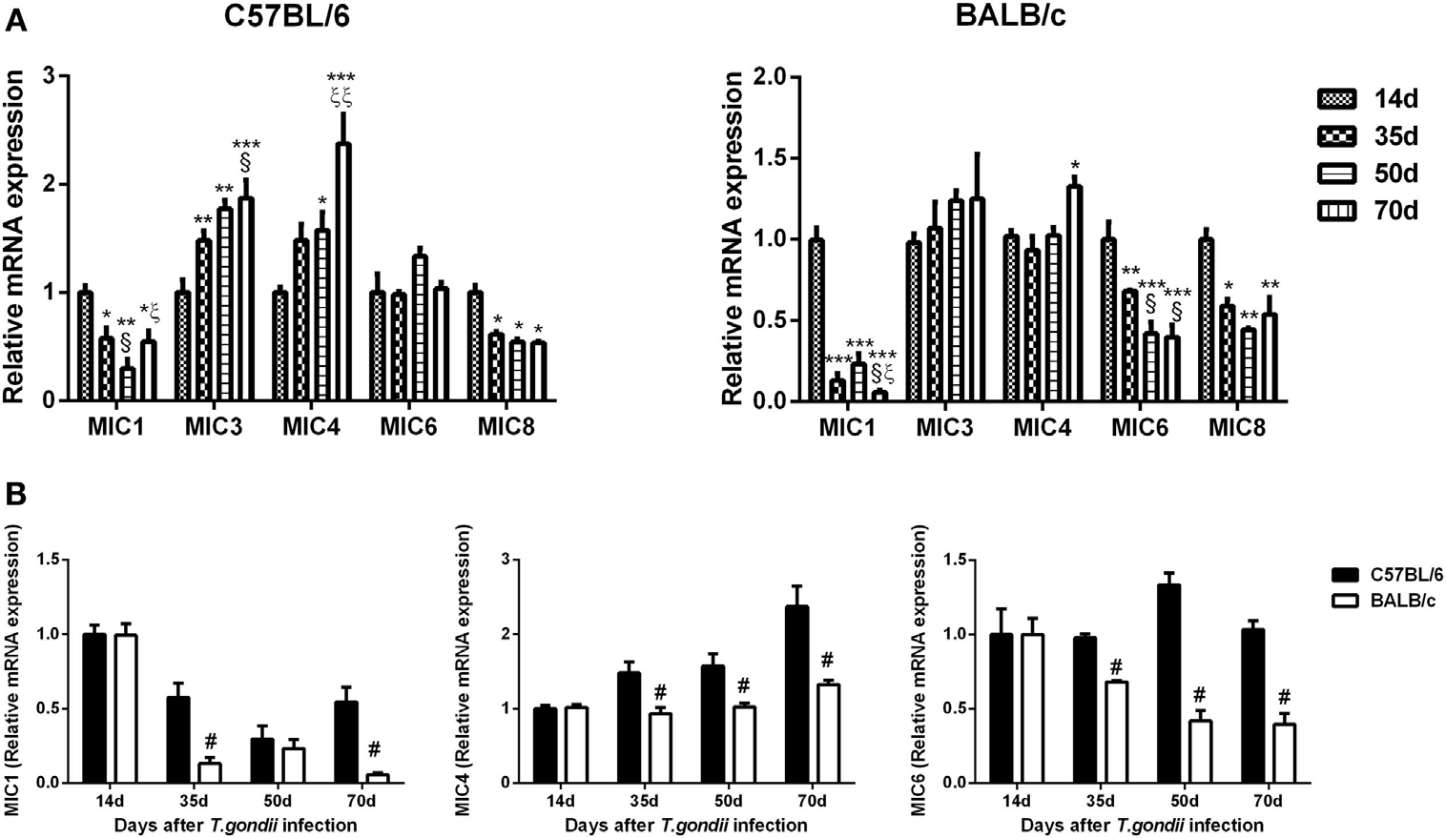
Relative mRNA expressions of TgMIC1, TgMIC4, TgMIC6, TgMIC3, and TgMIC8 in the brain tissues of *Toxoplasma gondii* Pru strain-infected C57BL/6 and BALB/c mice were detected by using quantitative real-time reverse transcription-polymerase chain reaction. **(A)** TgMICs expressions in the brains of C57BL/6 and BALB/c mice. **(B)** Comparison of TgMIC1, TgMIC4, and TgMIC6 levels in the brains of C57BL/6 and BALB/c mice. Transcript level at day 14 post infection (p.i.) was taken as 1. Values are means from triplicate measurements, and data are presented as mean ± SD. There were four mice per group, and data are representative of those from two different experiments. *P < 0.05, **P < 0.01, and ***P < 0.001 vs 14 days p.i.; ^§^*P* < 0.05 vs 35 days p.i.; ^ξ^*P* < 0.05 and ^ξξ^*P* < 0.05 vs 50 days p.i.; ^#^*P* < 0.05 vs C57BL/6 mice.

Compared with day 14 p.i., TgMIC1 levels were significantly decreased in the brains of both *T. gondii* Pru strain-infected C57BL/6 and BALB/c mice at days 35, 50, and 70 p.i.; TgMIC3 levels were significantly increased in C57BL/6 mice at days 35, 50, and 70 p.i.; TgMIC4 levels were significantly increased in C57BL/6 mice at days 50 and 70 p.i. and significantly increased in BALB/c mice at day 70 p.i. TgMIC6 levels were significantly decreased in BALB/c mice at days 35, 50, and 70 p.i., and TgMIC8 levels were significantly decreased in both C57BL/6 and BALB/c mice at days 35, 50, and 70 p.i. (Figure 6A). Compared with BALB/c mice, there were significantly higher levels of TgMIC1 at days 35 and 70 p.i. (*P* < 0.05) and significantly higher levels of TgMIC4 and TgMIC6 at days 35, 50, and 70 p.i. in the brains of C57BL/6 mice (*P* < 0.05) (Figure 6B).

### Correlations Between Gal-3/Ga-9 and Th1/Th2/M1/M2 Cytokines in the Brains of *T. gondii*-Resistant BALB/c and *T. gondii*-Susceptible C57BL/6 Mice

**Figure 7:**
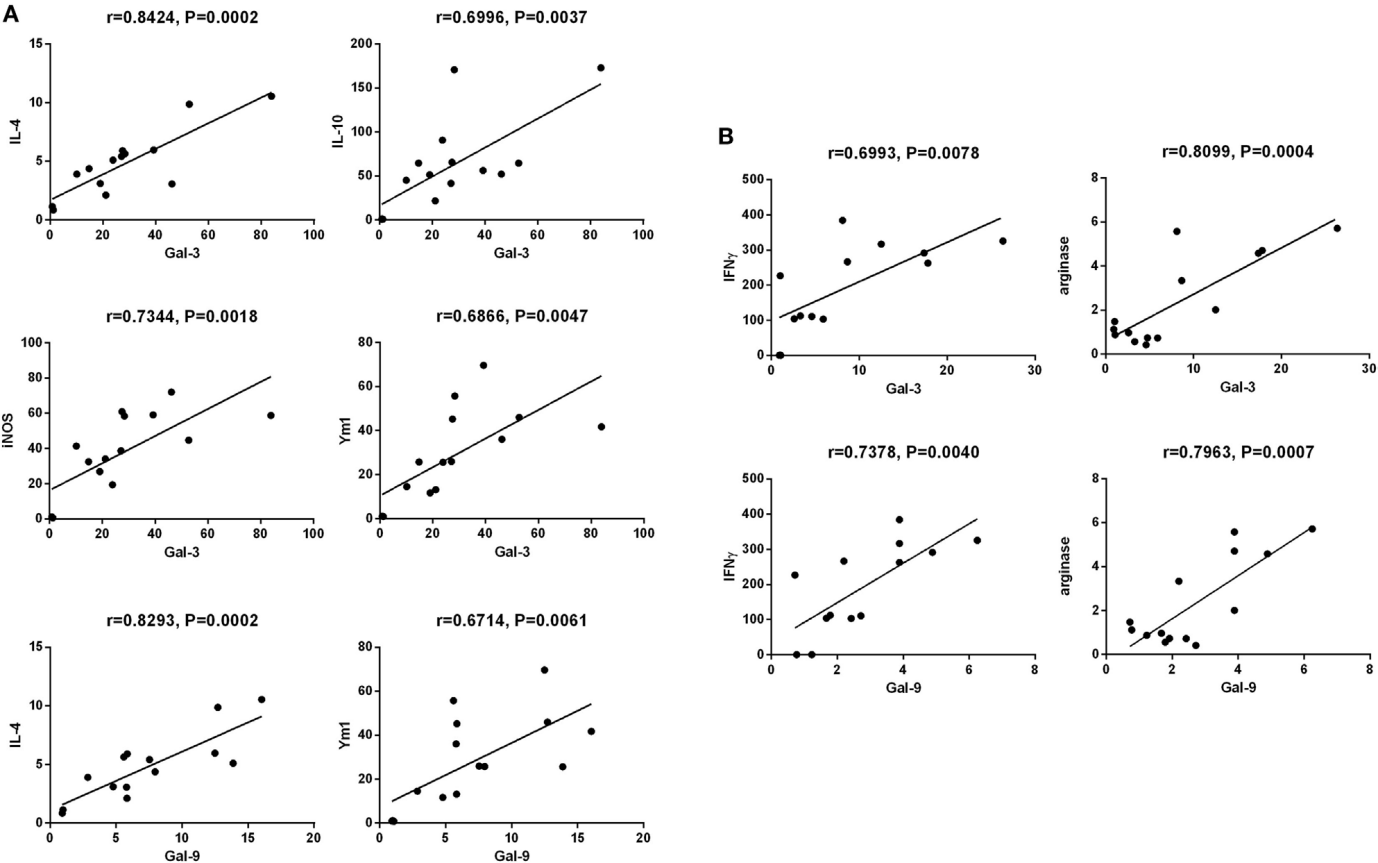
Correlation analysis between Gal-3 and T helper 1 (Th1)/Th2/M1/M2 cytokines as well as between Gal-9 and Th1/Th2/M2 cytokines in the brain tissues of *Toxoplasma gondii* Pru strain-infected C57BL/6 and BALB/c mice (*n* = 16). **(A)** Significant correlations between Gal-3 and IL-4/IL-10/iNOS/Ym1 as well as between Gal-9 and IL-4/Ym1 existed in the brains of *T. gondii*-infected C57BL/6 mice. **(B)** Significant correlations between Gal-3 and IFNγ/Arg1 as well as between Gal-9 and IFNγ/Arg1 existed in the brains of *T. gondii*-infected BALB/c mice. The *r* value generates for the theoretical line of best fit, and the *P* value indicates the significance of the correlation.

The correlations between mRNA levels of Gal-3/Gal-9 and Th1/Th2/M1/M2 in the brains of *T. gondii* Pru strain-infected C57BL/6 and BALB/c mice were evaluated, herein only significant correlations were shown. There were significant correlations between the mRNA levels of Gal-3 and IL-4 (*r* = 0.8424, *P* = 0.0002), Gal-3 and IL-10 (*r* = 0.6996, *P* = 0.0037), Gal-3 and iNOS (*r* = 0.7344, *P* = 0.0018), Gal-3 and Ym1 (*r* = 0.6866, *P* = 0.0047), Gal-9 and IL-4 (*r* = 0.8293, *P* = 0.0002), and Gal-9 and Ym1 (*r* = 0.6714, *P* = 0.0061) in *T. gondii* Pru strain-infected C57BL/6 mice (Figure 7A). However, there were significant correlations between the mRNA levels of Gal-3 and IFNγ (*r* = 0.6993, *P* = 0.0078), Gal-3 and Arg1 (*r* = 0.8099, *P* = 0.0004), Gal-9 and IFNγ (*r* = 0.7378, *P* = 0.0040), and Gal-9 and Arg1 (*r* = 0.7963, *P* = 0.0007) in *T. gondii* Pru strain-infected BALB/c mice (Figure 7B). Taken together, in C57BL/6 mice, significant positive correlations existed between Gal-3 and IL-4/IL-10/iNOS/Ym1 as well as between Gal-9 and IL-4/Ym1; whereas in BALB/c mice, significant positive correlations existed between Gal-3 and IFNγ/Arg1 as well as between Gal-9 and IFNγ/Arg1.

## Discussion

When *T. gondii* parasites infect the host, the cysts can exist predominantly in the brain tissue for lifetime, and an immunocompetent host will establish a strong and persistent Th1-biased cell-mediated immunity to resist cyst reactivation and the consequences of TE (4). However, there is a remarkable difference in susceptibility to the infection of *T. gondii* among inbred strains of mice. After peroral infection with *T. gondii* ME49 strain, C57BL/6 mice all died whereas BALB/c mice all survived (32). So far, the immune responses differing between TE-resistant and TE-susceptible hosts are not fully understood. In this study, genetically susceptible C57BL/6 and resistant BALB/c mice were perorally infected with *T. gondii* Pru strain, and significantly more severe histopathological damage (inflammation and necrosis) were found in the brains of C57BL/6 mice in comparison of those of BALB/c mice at all the times during the observations (e.g., at days 14, 35, 50, and 70 p.i.). The levels of mRNA transcripts of both tachyzoite-specific SAG1 and bradyzoite-specific BAG1 genes were significantly higher in the brains of C57BL/6 mice than those of BALB/c mice at days 35, 50, and 70 p.i. It has been reported that following *T. gondii* ME49 strain infection, C57BL/6 mice showed an intense and progressive inflammatory alteration in the CNS, while BALB/c mice showed slight inflammatory reaction in the CNS (33). After infection with low virulent *T. gondii* DX strain, C57BL/6 mice presented higher tachyzoite and bradyzoite loads than those of BALB/c mice (34). Our data were in accordance with the previous studies.

Microglia activation is recognized as the hallmark of neuroinflammation. Microglial cells are the primary source for inflammatory mediators. Resident microglial cells play a critical role in TE, producing essential pro- and anti-inflammatory cytokines such as IL-1β, IL-10, TNFα, IL-12, and IL-15 (35–37). In this study, we found that microglial cell numbers in the brains of C57BL/6 mice were significantly higher than those of BALB/c mice at days 35, 50, and 70 p.i.; however, the number of microglial cells was significantly higher in BALB/c mice than that of C57BL/6 mice at day 14 p.i. Our data suggest that resident microglia are activated earlier in BALB/c mice, which may be essential for control of the parasite in the early infective stage in *T. gondii*-resistant BALB/c mice; whereas increased microglial activation remains longer in C57BL/6 mice, which may be required for establishing chronic TE in *T. gondii*-susceptible C57BL/6 mice.

It has been reported that activated microglial cells range from the pro-inflammatory M1 phenotype to the alternative/M2 phenotype and play neuroprotective or neurodetrimental roles (38). Therefore, identifying microglia phenotypes is critical for understanding the role of microglia in the pathogenesis of TE. In this study, we found that alterations in M1 and M2 phenotypes differed between the two models. In *T. gondii* Pru strain-infected BALB/c mice, both M1 (iNOS) and M2 (Ym1) phenotypic markers were significantly increased in the early infective stage (at day 14 or 35 p.i.); while in C57BL/6 mice, both M1 (iNOS) and M2 (Ym1) phenotypic markers were significantly increased in the late infective stage (at days 50 and 70 p.i.) and M2 marker (Arg1) was significantly increased at all the times during the study. M1 macrophages are critical for host defense against intracellular pathogens and have roles in antitumor immunity and autoimmune inflammation, whereas M2 macrophages are protective against helminth parasites and are important regulators of the wound healing response, tissue homeostasis, and adiposity (39). Inhibition of iNOS exacerbates chronic TE in *T. gondii*-susceptible C57BL/6 mice but does not lead to reactivation of latent TE in *T. gondii*-resistant BALB/c mice (34). CBA/Ca mice are susceptible to the development of TE. An *in vitro* study demonstrated that microglia from CBA/Ca mice show decreased production of NO and decreased inhibition of *T. gondii* replication after stimulation with lipopolysaccharide or IFNγ plus TNFα compared with microglia from BALB/c mice (40). Our data demonstrated that both M1 (iNOS) and M2 (Ym1 and Arg1) responses may play a role during chronic TE in *T. gondii*-susceptible C57BL/6 mice.

*Toxoplasma gondii* infection induces Th1-biased immune response, which is critical for the prevention of reactivation of TE (41). In this study, although the mRNA levels of Th1-associated cytokines (IFNγ and IL-1β) and Th2-associated cytokines (IL-4 and IL-10) were increased in the brain tissues of both C57BL/6 and BALB/c mice infected with *T. gondii* Pru strain, susceptible C57BL/6 mice presented a dominant Th1 response characterized by high expression of IFNγ at all the times after infection, accompanied by stronger neuroinflammatory outcomes. Our data suggested that the delayed M1 and M2 microglial activation and increased IFNγ expression in C57BL/6 mice after *T. gondii* Pru strain infection may be a part of the reason that C57BL/6 mice are more susceptible than BALB/c mice during TE.

Galectins have recently been demonstrated to play vital roles in host–pathogen interaction (42). Galectins are important modulators participating in homeostasis of the CNS and neuroinflammation; the major galectins expressed in the CNS are Gal-1, Gal-3, Gal-4, Gal-8, and Gal-9 (18). In this study, we compared the dynamic gene expressions of Gal-1, Gal-3, Gal-4, Gal-8, and Gal-9 in the brains between C57BL/6 and BALB/c mice infected with *T. gondii* Pru strain, only Gal-3 and Gal-9 were highly expressed in the brains of both C57BL/6 and BALB/c mice. C57BL/6 mice presented significantly higher mRNA expressions of Gal-3 and Gal-9 than those of BALB/c mice at days 35, 50, and 70 p.i. Gal-3 and Gal-9 are known pro-inflammatory mediators and regulators of apoptosis (29). Gal-9 is produced by activated astrocytes (43), functions as an astrocyte–microglia communication signal and promotes cytokine production, such as TNF, from microglia (44). After infection with ME49 strain of *T. gondii*, gal3^−/−^ mice exhibits a higher parasite burden, delayed inflammatory response in the CNS, and significantly higher concentrations of IL-12p40 and IFNγ in the sera compared with those of gal3^+/+^ mice (45). Gal-3 is required for resident microglia activation and proliferation in response to ischemic injury in a mouse model (46). Our data demonstrated that both Gal-3 and Gal-9 are important factors in TE-susceptible C57BL/6 and TE-resistant BALB/c mice infected with *T. gondii* Pru strain. In addition, the inflammatory response is more pronounced in the brains of C57BL/6 mice, which are corresponded well with the increased numbers of Iba1-positive resident microglia as well as increased Gal-3 and Gal-9 expressions in C57BL/6 mice. Furthermore, we evaluated the correlations between the gene expressions of Gal-3/Gal-9 and the levels of Th1 and Th2 cytokines, and M1- and M2-associated cytokines in the brains after *T. gondii* Pru strain infection. Positive correlations were found in the mRNA levels between Gal-3 and IL-4/IL-10/iNOS/Ym1 as well as between Gal-9 and IL-4/Ym1 in C57BL/6 mice; whereas positive correlations were found between Gal-3 and IFNγ/Arg1 as well as between Gal-9 and IFNγ/Arg1 in BALB/c mice. These data suggest that Gal-3 is related to Th2 and M1/M2 immune responses while Gal-9 is related to Th2 and M2 immune responses in *T. gondii*-infected C57BL/6 mice. Indeed, both Gal-3 and Gal-9 are related to Th1 and M2 immunity in BALB/c mice with chronic *T. gondii* infection. Our data suggested that Gal-3 and Gal-9 may involve in different immune responses to *T. gondii* Pru strain infection in the two lineages of mice.

Proteins secreted from apicomplexan MICs play important roles in the parasite adhesion and invasion of the host cells (47). MICs, which have been identified with lectin domains, support several key cellular processes including gliding motility, active cell invasion and migration through cells, biological barriers, and tissues (47). Our data showed that C57BL/6 mice expressed significantly higher levels of TgMIC1, TgMIC4, and TgMIC6 at days 35, 50, or 70 p.i. than those of BALB/c mice after *T. gondii* Pru strain infection. Therefore, TgMICs may be expressed differently in the two strains of mice with different genetic background. TgMIC1–4–6 complex contributes to host cell recognition and attachment *via* the action of TgMIC1 as well as contributes to the virulence of *T. gondii* in mice (48). Our data indicate that the different expression levels of TgMIC1, TgMIC4, and TgMIC6 in the two strains of mice may be associated with the different outcomes in *T. gondii* Pru strain*-*infected C57BL/6 and BALB/c mice.

In conclusion, this study has provided evidences that Gal-3 and Gal-9 may play a critical role in the regulation of M1, M2, Th1, and Th2 cytokines in the hosts with TE. Our data demonstrated that significant different mRNA expressions of Gal-3 and Gal-9 as well as microglial activation markers, cytokines, and TgMICs were found between C57BL/6 and BALB/c mice after *T. gondii* Pru strain infection. Whether these differences are related to the phenomenon that C57BL/6 mice are susceptible while BALB/c mice are resistant to the development of TE needs to be further investigated.

## Ethics Statement

This study was carried out in accordance with the recommendations of the requirements of the Animal Ethics Committee at Sun Yat-sen University. The protocol was approved by the Animal Ethics Committee at Sun Yat-sen University.

## Author Contributions

FL designed experiments, wrote, and edited the manuscript. JL conducted experiments, analyzed data, and wrote the manuscript draft. SH revised and edited the manuscript.

## Conflict of Interest Statement

The authors declare that the research was conducted in the absence of any commercial or financial relationships that could be construed as a potential conflict of interest.
